# *Inquilinus limosus* and Cystic Fibrosis

**DOI:** 10.3201/eid1406.071355

**Published:** 2008-06

**Authors:** Fadi Bittar, Anne Leydier, Emmanuelle Bosdure, Alexandre Toro, Martine Reynaud-Gaubert, Stéphanie Boniface, Nathalie Stremler, Jean-Christophe Dubus, Jacques Sarles, Didier Raoult, Jean-Marc Rolain

**Affiliations:** * Université de la Méditerranée, Marseille, France; † Hôpital Timone, Marseille, France; ‡ Hôpital Sainte Marguerite, Marseille, France

**Keywords:** Inquilinus limosus, cystic fibrosis, colistin resistance, real-time PCR, letter

**To the Editor:** *Inquilinus limosus*, a new multidrug-resistant species, was reported in 1999 as an unidentified gram-negative bacterium in a lung transplant patient with cystic fibrosis (CF) (*1*). This species was later characterized by the description of 7 new isolates of *I. limosus* and 1 isolate of *Inquilinus* sp (*2*). Infections and colonizations by *I. limosus* have been documented mainly in adolescent or adult patients with CF. To date, 8 clinical cases have been described in Germany (*3*,*4*), 1 case in the United States (*1*), 5 cases in France (*5*), and 1 case in the United Kingdom (*6*) (Table). Only 1 isolate of *Inquilinus* sp. has been recovered from blood samples of a patient without CF who had prosthetic valve endocarditis (*7*).

**Table:** Clinical and epidemiologic features of cystic fibrosis (CF) patients with Inquilinus limosus*

Case no.	Age, y/sex	Lung transplant	Positive samples	Clinical manifestation (first isolation)	Growth on MacConkey agar	Growth on selective agar (d)	Phenotypic identification†	Other associated pathogens	Reference
1	22/F	Yes	Lung explant, BAL, sputum	Pneumonia	Poor	ND	AR	PA, PM	(*1*)
2	17/M	No	Sputum	Stable	No	Yes (6 d)	SP	SA, PA, CA	(*3*)
3	14/F	No	Sputum	Stable	No	Yes (5 d)	SP	PA, AF, CA	(*3*)
4	12/M	No	Sputum	Stable	ND	Yes (ND)	SP	PA, SM, SA, AX	(*5*)
5	13/F	No	Sputum	Exacerbation	ND	Yes (ND)	SP	PA, SA, AF	(*5*)
6	8/M	No	Sputum	Stable	ND	Yes (ND)	SP	PA	(*5*)
7	10/M	No	Sputum	Stable	ND	Yes (ND)	SP	None	(*5*)
8	18/M	No	Sputum	Exacerbation	ND	Yes (ND)	AR	PA, SA, AF	(*5*)
9	16/F	No	Sputum	Severe exacerbation	ND	ND	PA	PA	(*4*)
10	19/M	No	Sputum	Stable	ND	ND	ND	PA	(*4*)
11	17/F	No	Sputum	Exacerbation	ND	ND	ND	PA, CA, AF	(*4*)
12	20/F	No	Sputum	Exacerbation	ND	ND	ND	PA, SA, CA, AF	(*4*)
13	17/F	No	Sputum	Stable	ND	ND	ND	PA, SA, SM, CA, AF	(*4*)
14	35/M	No	Sputum	Respiratory decline	ND	ND	PA	PA, SM, SMA	(*4*)
15	17/F	No	Sputum	Stable	No	Yes (4 d)	SP	CA	This study
16	2/M	No	Sputum	Productive cough	No	Yes (3 d)	SP	SA, HI	This study
17	21/M	No	Sputum	Exacerbation	No	Yes (3 d)	AR	PA, AF	This study
18	15/M	No	Sputum	Fever and thoracic pain	No	Yes (3 d)	AR	SA	This study

*BAL, bronchoalveolar lavage; ND, not determined; AR, *Agrobacterium radiobacter*; PA, *Pseudomonas aeruginosa*; PM, *Proteus mirabilis*; SP, *Sphingomonas paucimobilis*; SA, *Staphylococcus aureus*; CA, *Candida albicans*; AF, *Aspergillus fumigatus*; SM, *Stenotrophomonas maltophilia;* AX, *Achromobacter xylosoxidans*; SMA, *Serratia marcescens;* HI, *Haemophilus influenzae.* †Phenotypic identification was obtained by using the BIOLOG GN MicroPlate assay (BIOLOG Inc., Hayward, CA, USA) for case 1 and the API 20NE kit system (bioMérieux, Marcy l’Etoile, France) for cases 2–8 and 15–18.

Because this bacterium is not recorded in all commercial identification system databases currently available, a longitudinal study for *I. limosus* detection with a new real-time PCR assay with a Taqman probe (Applied Biosystems, Foster City, CA, USA), that targets the 16S rRNA gene, has been developed and compared with the culture isolation. Primers il1d (5′-TAATACGAAGGGGGCAAGCGT-3′) and il1r (5′-CACCCTCTCTTGGATTCAAGC**-**3′) and probe ilProbe (6FAM-GGTTCGTTGCGTCAGATGTGAAAG-TAMRA), which were used in this study, were designed on the basis of multisequence alignment of all *I. limosus* 16S rDNA sequences available in the GenBank database.

To confirm specificity, the primers and probe were checked by using the BLAST program (www.ncbi.nlm.nih.gov/blast/Blast.cgi) and also by using suspension of several bacteria recovered habitually in patients with CF. For sensitivity of the Taqman PCR assay (Applied Biosystems), the minimal CFU detectable was 2 CFU/PCR. From January 2006 through June 2007, 365 sputum samples recovered from 84 children and 61 adults with CF and 71 sputum samples recovered from 54 patients without CF were screened blindly for *I. limosus*. By using our real-time PCR, we detected 9 *I. limosus*-positive samples from 4 patients with CF (Table); 8 of these samples were also culture positive. However, all sputum samples from patients without CF were negative. In 1 patient (Table, case 17), *I. limosus* was detected by using real-time PCR 3 months before the culture was positive. Retrospectively, the patient’s medical file was rechecked and his clinical respiratory condition worsened briefly at that stage, which indicates an infection by this bacterium. Thus, in our study, the incidence of *I. limosus* was 2.8% (4.9% for adults with CF and 1.2% for children with CF). The incidence of *Burkholderia cepacia* complex during the same period and in the same patients was 2.1% (3 adults with CF were positive, data not shown).

The genus *Inquilinus* belongs to the α-*Proteobacteria*; the genus *Azospirillum* is the most closely related bacteria (*2*). This cluster of bacteria contains several strains that are able to grow under saline conditions and in biofilms (*8*,*9*). The mucoid phenotype of *I. limosus* may contribute to its colonization and resistance to many antimicrobial drugs. Recently, the exopolysaccharides (EPS) produced by *I. limosus* were studied. The authors indicated that *I. limosus* produces mainly 2 EPSs that exhibit the same charge per sugar residue present in alginate, the EPS produced by *Pseudomonas aeruginosa* in patients with CF. This similarity may be related to common features of the EPS produced by these 2 opportunistic pathogens that are related to lung infections (*10*). Transmission of *I. limosus* between patients with CF is not known, but in the report from Chiron et al., 1 of the 5 patients with *I. limosus* had a brother who had never been colonized with this bacterium despite living in the same home (*5*). Schmoldt et al. reported that 3 patients were treated in the same outpatient CF clinic during overlapping time periods and each patient was infected/colonized by an individual *I. limosus* clone, which suggests that there was no transmission among these patients (*4*). This bacterium has been recovered mainly from sputum of adolescents (mean age 17 ± 6.47 years, range 8–35), except in our study with a 2-year-old boy, which suggests that this emerging bacterium may be hospital acquired, as recently suggested (*7*). Because this bacterium is multiresistant to several antimicrobial drugs, particularly colistin, which is widely used for treatment for *P. aeruginosa* colonization (as was the case for our 4 patients), we hypothesize that this bacterium is selected during the evolution of the disease.

We have developed a real-time PCR molecular method that is faster and easier than amplification-sequencing for prompt detection and accurate identification of *I. limosus* with good specificity and sensitivity. By using this screening assay, we identified 4 additional cases of patients with CF who were also infected with this bacterium, including a 2-year-old child. In addition, by using this technique, we were able to detect *I. limosus* in a patient with deteriorated respiratory function 3 months before the culture-based isolation, indicating that a low bacterial load, insufficient for being isolated in culture, can be detected by PCR in the lower respiratory tract of patients with CF.
